# Shifts in Abundance and Diversity of Soil Ammonia-Oxidizing Bacteria and Archaea Associated with Land Restoration in a Semi-Arid Ecosystem

**DOI:** 10.1371/journal.pone.0132879

**Published:** 2015-07-14

**Authors:** Zhu Chen, Wenliang Wu, Xiaoming Shao, Li Li, Yanbin Guo, Guochun Ding

**Affiliations:** 1 College of Biological Sciences, China Agricultural University, Beijing, China; 2 College of Resources and Environmental Science, China Agricultural University, Beijing, China; 3 Beijing Key Laboratory of Biodiversity and Organic Farming, China Agricultural University, Beijing, China; University of Vigo, SPAIN

## Abstract

The Grain to Green Project (GGP) is an unprecedented land restoration action in China. The project converted large areas (ca 10 million ha) of steep-sloped/degraded farmland and barren land into forest and grassland resulting in ecological benefits such as a reduction in severe soil erosion. It may also affect soil microorganisms involved in ammonia oxidization, which is a key step in the global nitrogen cycle. The methods for restoration that are typically adopted in semi-arid regions include abandoning farmland and growing drought tolerant grass (*Lolium perenne* L.) or shrubs (*Caragana korshinskii* Kom.). In the present study, the effects of these methods on the abundance and diversity of ammonia-oxidizing bacteria (AOB) and ammonia-oxidizing archaea (AOA) were evaluated via quantitative real-time PCR, terminal restriction fragment length polymorphism and clone library analysis of *amoA* genes. Comparisons were made between soil samples from three restored lands and the adjacent farmland in Inner Mongolia. Both the abundance and community composition of AOB were significantly different between the restored lands and the adjacent control. Significantly lower nitrification activity was observed for the restored land. Clone library analysis revealed that all AOB *amoA* gene sequences were affiliated with *Nitrosospira*. Abundance of the populations that were associated with *Nitrosospira* sp. Nv6 which had possibly adapted to high concentrations of inorganic nitrogen, decreased on the restored land. Only a slight difference in the AOB communities was observed between the restored land with and without the shrub (*Caragana korshinskii* Kom.). A minor effect of land restoration on AOA was observed. In summary, land restoration negatively affected the abundance of AOB and soil nitrification activities, suggesting the potential role of GGP in the leaching of nitrates, and in the emission of N_2_O in related terrestrial ecosystems.

## Introduction

China implemented the Grain to Green Program (GGP) in 1897 counties of 25 provinces, including Inner Mongolia, to increase vegetation coverage [1]. By the end of 2012, approximately 9.7 million hectares of cropland had been converted to forest or grassland [2]. This greatly improved soil conditions across China through decreasing soil erosion [3], and increasing soil organic carbon content [4]. There are three typical restoration methods in Inner Mongolia: abandoning farmland, growing grass (*Lolium perenne* L.), and growing shrubs (*Caragana korshinskii* Kom.). Both *Lolium perenne* L. and *Caragana korshinskii* Kom. are drought-tolerant plants suitable for land restoration in arid or semi-arid regions [5]. Converting farmland into grassland or scrubland alters cover vegetation, which determines the quality and quantity of root exudates serving as nutrients for microorganisms [6]. Changes in fertilization, tillage, irrigation and other agricultural practices possibly altered soil physicochemical properties which could influence soil microbial communities as well [7]. The influences of land use on the diversity and community composition of soil microorganisms have been evaluated in several studies [8–10]. Despite numerous environmental benefits, the effects of land restoration on the soil nitrogen cycle, as well as on participating microorganisms, remain poorly understood.

Aerobic ammonia oxidation mediated by bacteria within *β-* and *γ- Proteobacteria* [11] and archaea within *Thaumarchaeota* [12, 13] is the rate-limiting step in nitrification, which influences plant nitrogen availability, the leaching of nitrates into ground water, greenhouse gas N_2_O emission [14]. Various studies attempted to discern the relative contribution of ammonia-oxidizing bacteria (AOB) and ammonia-oxidizing archaea (AOA) in nitrification across different ecological niches [15, 16]. In terrestrial ecosystems, the abundance of AOA frequently exceed that of AOB, which points to the potential role of AOA in nitrification [17]. The abundance ratios of AOA to AOB in a number of studies negatively correlated with pH values [16, 18, 19], and ex-situ culture of AOB has failed in acidic conditions [20]. This indicates the importance of AOA to nitrification in acidic soils. In the study of Lu et al. [21], urea fertilization was found to stimulate nitrification activity and the growth of AOA from the marine Group 1.1a-associated lineage in the acidic soil. While in an alkaline soil the abundance and composition of AOB, not AOA, was significantly impacted by long-term fertilization [22]. In addition to pH, the ammonia concentration also determined the differential growth of AOA and AOB, and growth of AOB could be promoted at high ammonia levels [23]. In dryland ecosystems, the availability of water played an important role in the activities of ammonia oxidizers [24], and AOB may responded faster than AOA to the addition of water [25].

In this study, we hypothesized that land restoration would affect both AOA and AOB and the effects varied between different restoration methods. We then compared the nitrification activity and the abundance and diversity of the two ammonia oxidizers between degraded farmland and three adjacent restoration lands using the three typical methods used in semi-arid Inner Mongolia.

## Materials and Methods

### Field site description and soil sampling design

The study area is in Wuchuan County (40°59'N, 110°33'E) in Inner Mongolia Province, China. This area is characterized by typical semi-arid characteristics with a temperate continental climate and with mean annual temperature at 2.7°C and mean annual precipitation at 250 mm– 400 mm, over 80% of which is between June and September [26]. The soil is classified as Haplic Calcisol (FAO/UNESCO soil classification system). The farmland in Wuchuan was mainly converted from pasture during the 1950s [27], with wheat, sunflower and potato as main crops. The GGP has been implemented here since 2002 using three major restoration methods. Farmlands were (1) abandoned and allowed to be naturally recolonized with indigenous species, (2) sown with *Lolium perenne* L., and (3) planted with *Caragana korshinskii* Kom. This resulted in three land cover types including grassland with indigenous species (e.g. *Agropyron cristatum* L. Gaertn., *Leymus chinensis* Trin. Tzvel., *Heteropappus altaicus* Willd. Novopokr. and *Artemisia frigida* Willd.), grassland dominated by *Lolium perenne* L., and scrubland dominated by *Caragana korshinskii* Kom. According to our survey (data not published), 56.9 thousand ha of land had been converted to forest or grassland in Wuchuan County until 2013. Four land cover types were selected, including farmland (sunflower-potato-wheat three-year rotation, one crop per year, with urea at 300 kg N ha^-1^ per year and superphosphate at 150 kg P_2_O_5_ ha^-1^ per year), abandoned farmland, *Lolium perenne* L. land, and *Caragana korshinskii* Kom. land, with the latter three converted from farmland in 2002. No specific permits were required for sampling. The location is not protected in any way, and there are no impacts on endangered species. Soil samples were taken on 9 July 2013 during the optimal growth period of native vegetation. Five replicates per cover were sampled and each replicate consists of six cores of soil from 0–20 cm depth. The different plots were 1.5–5 km apart from each other. Samples were then transported inside an ice box to the laboratory, sieved through 2 mm mesh, and stored under -80°C condition before DNA extraction.

### Soil physico-chemical properties

All soil parameters were analyzed in the laboratory using standard methods. Soil pH was determined in 1:2.5 (wt/vol) diluted water suspension [28]. Soil moisture content was measured by drying fresh soil at 105°C for 24 hours. Phosphate was measured according to Olsen et al. [29]. Ammonia and nitrate were extracted with 1M KCl and measured using a continuous flow analyzer (Bran-Luebbe, Norderstedt, Germany). Soil organic carbon and total N were measured by CNS analyzer (Thermo Fischer scientific Inc., Sunnyvale, CA, USA). The soil chemical properties are listed in Table 1.

**Table 1:** Soil physico-chemical properties.

Cultivation Systems	FL	AFL	LL	CL
Water content (%)	8.71±0.21a	8.87±0.26a	8.42±0.32a	5.03±0.44b
pH (H_2_O)	8.11±0.03b	8.27±0.03a	8.26±0.04a	8.18±0.03ab
SOC (g kg^−1^)	9.46±0.14c	9.42±0.42bc	11.77±0.56a	10.93±0.17ab
TN (g kg^−1^)	0.97±0.04a	0.99±0.10a	1.07±0.06a	1.02±0.05a
C/N ratio	15.84±0.30b	19.63±1.04a	16.24±2.30b	11.55±0.07c
NH_4_^+^-N (mg kg^−1^)	8.83±0.57a	6.06±0.56b	7.65±0.54ab	5.38±1.12b
NO_3_^−^-N (mg kg^−1^)	19.40±2.46a	4.82±0.61b	4.35±0.35b	4.60±1.37b
Phosphate (mg kg^−1^)	56.62±4.46a	35.11±2.52b	35.69±2.26b	34.66±0.53b

SOC, soil organic carbon, TN, total nitrogen, FL, farmland, AFL, abandoned farmland, LL, *Lolium perenne* L. land, CL, *Caragana korshinskii* Kom. land.

Mean ± standard error (n = 5). Significant differences (*p* < 0.05) between systems are shown with letters a, b or c.

### Potential nitrification rate

The potential nitrification rate (PNR) was determined via the chlorate inhibition method [30]. Fifty grams of soil sample were placed in the Erlenmeyer flask with 60 ml phosphate buffer solution (g l^-1^: NaCl, 8.0; KCl, 0.2; Na_2_HPO_4_, 0.2; NaH_2_PO_4_, 0.2; pH 7.4) with 1 mM (NH_4_)_2_SO_4_. Potassium chlorate with a final concentration of 10 mg l^-1^ was added to the flask to inhibit nitrite oxidation. The slurry was then shaken on an orbital shaker at 180 rpm for 24 h under 25°C to maintain an aerobic environment in the dark. The concentration of nitrite was measured at 6, 12, 18 and 24 h after incubation according to Shen et al. [22]. PNR was finally calculated from the rate of linear regression for nitrite concentrations with time.

### Soil DNA extraction and quantitative real-time PCR

Soil DNA was extracted from 0.25 g of soil using the PowerSoil DNA Isolation Kit (Mobio Laboratories Inc., Carlsbad, CA, USA) following the manufacturer's instructions. Primer pairs of *amoA*-1F/*amoA*-2R [31] and Cren*amoA*23f/Cren*amoA*616r [32] were used for quantitative real-time PCR (qPCR) analyses of AOB and AOA *amoA* genes, respectively. The qPCR assays were performed using an ABI 7500 Real-time PCR instrument (Applied Biosystems, Foster City, CA, USA). The reaction mixture (20 μl) contained 10 μl of SYBR Premix Ex Taq (Takara Biotechnology, Dalian, China), 1 μM of respective primer, 2 μg of BSA, and approximately 20 ng template or standard DNA. The specificity and efficiency of amplification was checked by melting curve analysis. Templates to generate standard curve were prepared by the serial dilution of purified plasmid DNA with *amoA* genes for AOB or AOA respectively.

### Terminal restriction fragment length polymorphism analysis of AOA and AOB

For terminal restriction fragment length polymorphism (tRFLP) analysis of the bacterial *amoA* genes, PCR amplification was performed using *amoA*-1F and *amoA*-2R [31] with forward primer 6-carboxyfluorescein (FAM) labeled. The thermal profile consisted of 5 min at 94°C, 35 cycles of 30 s at 94°C, 30 s at 57°C and 60 s at 72°C, and 10 min at 72°C for extension. The PCR product was checked by agarose gel electrophoresis and purified using PCR Clean-Up System (Axygen Scientific, Union City, CA, USA). The purified products were digested with the restriction enzyme *Msp* I (NEB, Beijing, China). For tRFLP analysis of the archaea *amoA* genes, the PCR amplification was performed using Cren*amoA*23f and Cren*amoA*616r [32] with forward primer FAM labeled. The terminal profile for PCR amplification included 5 min at 94°C, 35 cycles of 45 s at 94°C, 30 s at 60°C and 60 s at 72°C, and 10 min at 72°C. The PCR products were checked and purified similarly as bacterial *amoA* genes. The purified products of AOA were digested with *Mbo* I (NEB, Beijing, China). After digestion, the sample of AOB and AOA was mixed with formamide and the internal standard GeneScanTM-500 LIZ (Applied Biosystems, Foster City, CA, USA). The mixed solutions were size-separated using an ABI-PRISM 3030XL Genetic Analyzer (Applied Biosystems, Warrington, UK). The relative abundance of each peak was quantified with GeneMarker version 2.2 (http://www.softgenetics.com).

### Cloning, sequencing and phylogenetic analysis

*AmoA* genes of AOA and AOB were amplified using the same PCR conditions as the tRFLP analysis with non-FAM labeled primer. For each treatment, an equal volume of PCR products of five replicates were pooled to construct clone libraries. The purified PCR products were cloned into the *pEASY*-T1 vector (TransGen Biotech, Beijing, China). Thirty clones for each treatment were randomly selected for sequencing.

After truncating the primer regions, all sequences were subjected to BLAST-N and BLAST-X analyses to identify *amoA* gene sequences. Phylogenetic analysis of *amoA* genes was performed separately for AOA and AOB. The cloned gene sequences as well as known *amoA* genes were aligned and the phylogenetic tree was constructed using neighbor-joining method and tested by bootstrap analysis using MEGA version 4.0 [33]. The aligned sequences were assigned to operational taxonomic units (OTUs) by Mothur [34] at the 97% sequence similarity level. All the *amoA* gene sequences have been deposited in the GenBank under accession numbers KJ652991 to KJ653018 for AOB and KJ699396 to KJ699421 for AOA.

### Statistical analysis

Two-way ANOVA in conjunction with Duncan's protected least significant difference tests (*p* < 0.05) were performed to examine the significance of treatment effects on the abundance of AOA and AOB using a log-transformed copy number of *amoA* genes. Spearman's correlation coefficients were calculated to assess the significant relations between the gene copy numbers, PNA, and soil chemical parameters. Principal component analysis (PCA) and Cluster analysis were applied to compare the tRFLP profiles of AOA and AOB. Analysis of similarity (ANOSIM) [35] was applied to examine the significance of treatment effects on the tRFLP profiles of AOA and AOB. The bigger ANOSIM’s R value means the more difference between groups. Redundancy analysis (RDA) with Monte Carlo permutation test (499 permutations) was applied to test which environmental variables best explained the variation in AOA and AOB communities. Cluster analysis and ANOSIM were performed with the vegan package (http://cran.r-project.org/web/packages/vegan/index.html) of R (http://www.R-project.org). The multivariate analyses were performed with Canoco for Windows version 4.5 (Biometry, Wageningen, Netherlands).

## Results

### Soil physico-chemical properties

To compare soil physico-chemical characteristics between different treatments, total organic carbon and nitrogen, pH, ammonium, nitrate, phosphate, and water content were quantified. The pH values of soils from both grasslands were slightly but significantly higher than that of the cropland (Table 1). As expected, the concentrations of ammonium, nitrate and phosphate were lower in the restored land soils than that of in the cropland, in which additional fertilizer was applied annually (Table 1). However, two-way ANOVA analysis revealed non-significant differences for total nitrogen content amongst all treatments (Table 1). Soil organic carbon was higher in the restored land than in the cropland, with the exception of abandoned farmland (Table 1). The carbon-to-nitrogen ratio of abandoned farmland was significantly higher than that of cropland (Table 1). The lowest carbon-to-nitrogen ratio and water content were measured in land growing *Caragana korshinskii* Kom. (Table 1).

### PNR measurement and abundances of AOB and AOA in soils

**Fig 1:**
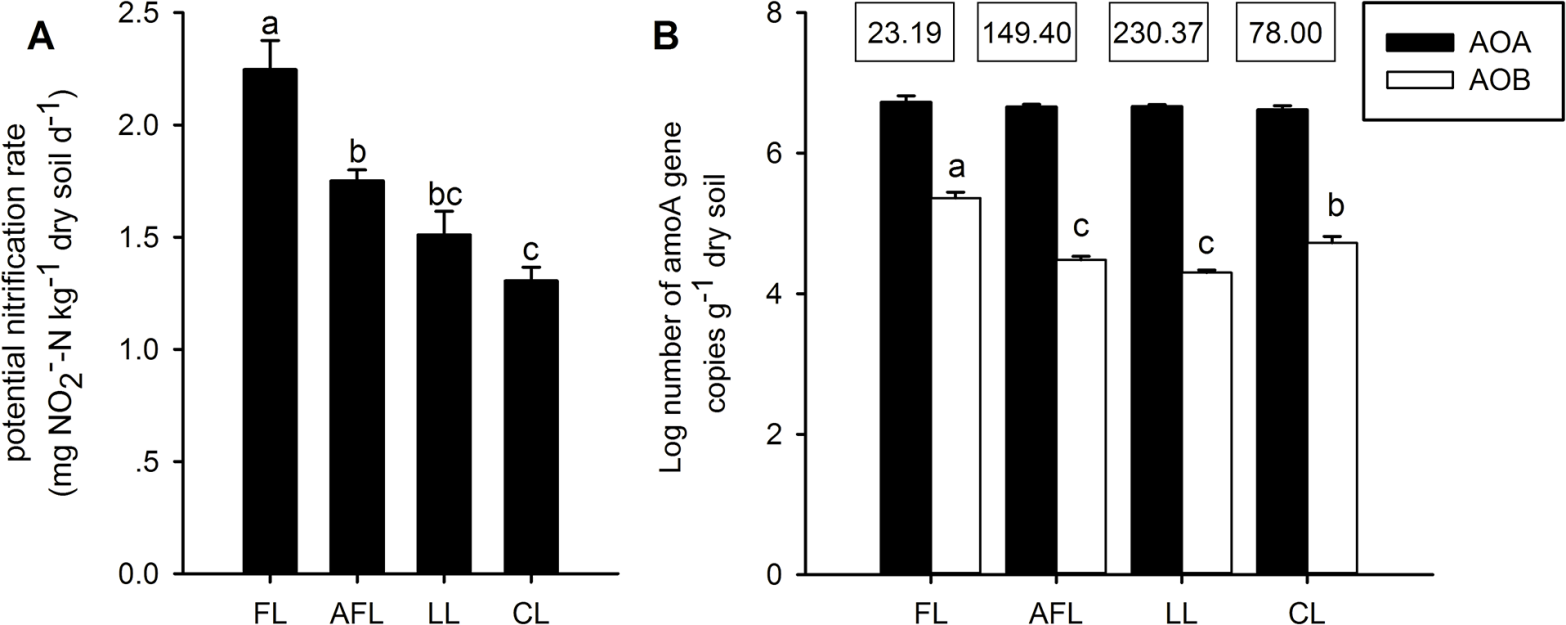
Potential nitrification rate (A), bacterial and archaea *amoA* genes abundances (B) in different land cover. FL, farmland, AFL, abandoned farmland, LL, *Lolium perenne* L. land, CL, *Caragana korshinskii* Kom. land. The *amoA* gene copy numbers were log-transformed. Values are mean ± standard error (n = 5). Means designated with different letters are significantly different at *p* < 0.05 based upon Duncan's protected least significant difference test. The ratios of AOA to AOB *amoA* gene copies are shown in the boxes above the chart.

To study the effect of land restoration on the nitrogen cycle, the overall activities and abundances of ammonia-oxidizing bacteria and archaea were explored. Potential nitrification rates were measured for soils from the cropland and restored lands (Fig 1A). The highest activity was detected for cropland, while the lowest was found for soil in the *Caragana korshinskii* Kom. land (Fig 1A). However, the difference of PNR in soil between *Caragana korshinskii* Kom. land and *Lolium perenne* L. land was non-significant (Fig 1A). Interestingly, PNR showed a positive correlation with soil water content (r = 0.629, n = 20, *p <*0.01) and NO_3_^—^N concentration (r = 0.719, n = 20, *p <* 0.001), while a negative correlation with total nitrogen (r = -0.480, n = 20, *p <* 0.05) and total organic carbon (r = -0.443, n = 20, *p <* 0.05).

The abundance of AOB and AOA were measured by quantitative PCR analysis of the *amoA* gene. The abundance of AOB was significantly higher in cropland than that of the restored land (Fig 1B). Surprisingly, the AOB abundance for abandoned farmland and *Lolium perenne* L. land was significantly lower than that of *Caragana korshinskii* Kom. land (Fig 1B), where the lowest PNR was detected (Fig 1A). Nevertheless, the PNR still positively correlated with the abundance of AOB *amoA* gene copies (r = 0. 736, n = 20, *p <* 0.01). Based on the qPCR analysis of *amoA*, AOA was more abundant than AOB in all land types (Fig 1B, as the AOA to AOB ratio were 23.19–230.37), but no significant differences of AOA *amoA* gene copy numbers were observed between any treatments (Fig 1B).

### Genetic profiling of bacterial and archaea *amoA* genes by tRFLP

To compare the community composition of soil ammonia oxidizing bacteria and archaea between the cropland and restored lands, the *amoA* gene amplicons of AOA and AOB were analyzed by tRFLP.

**Fig 2:**
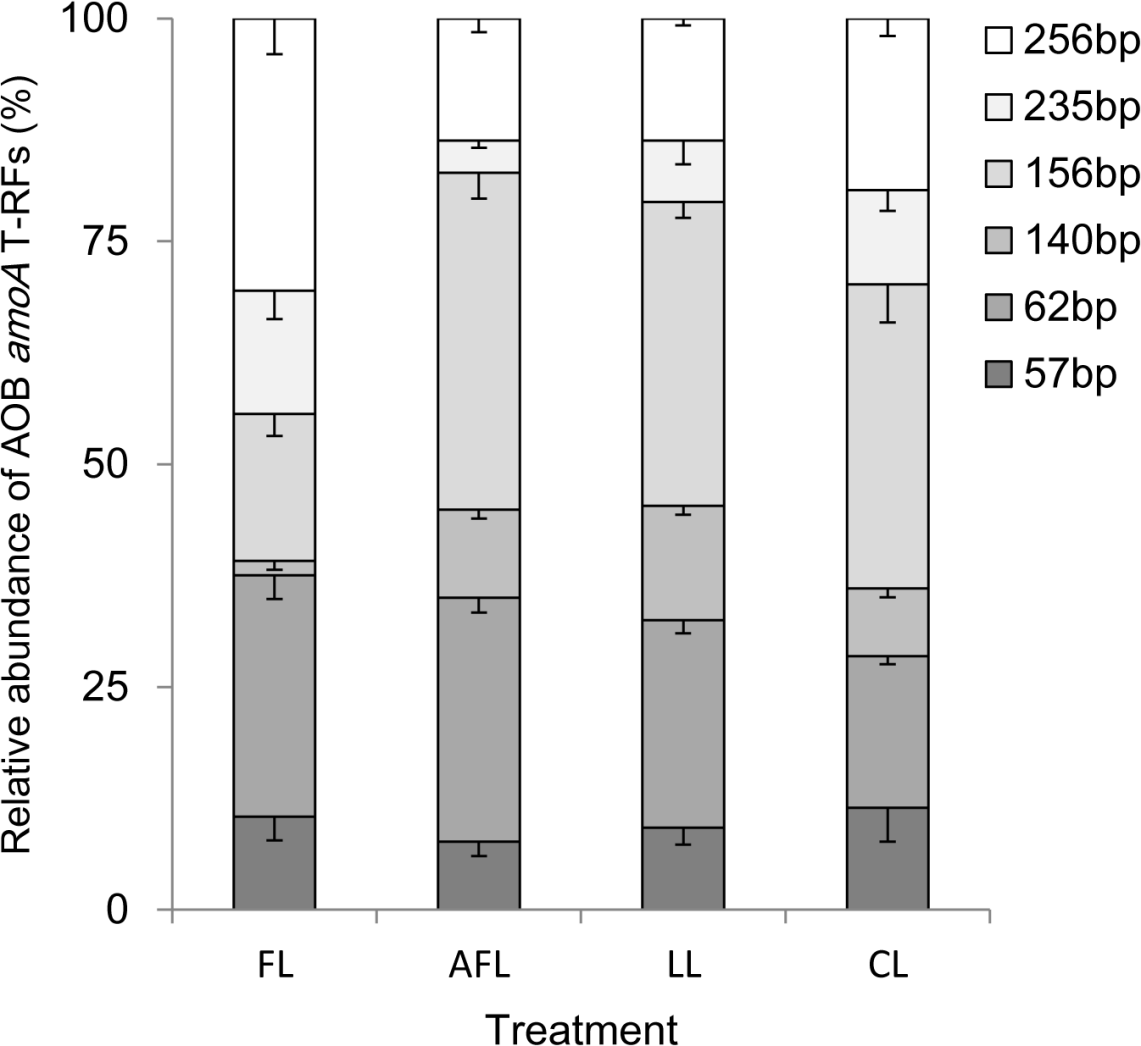
Terminal restriction fragment length polymorphism fingerprints of the bacterial *amoA* gene fragments in different land cover. Error bars indicate standard errors (n = 5). FL, farmland, AFL, abandoned farmland, LL, *Lolium perenne* L. land, CL, *Caragana korshinskii* Kom. land.

**Fig 3:**
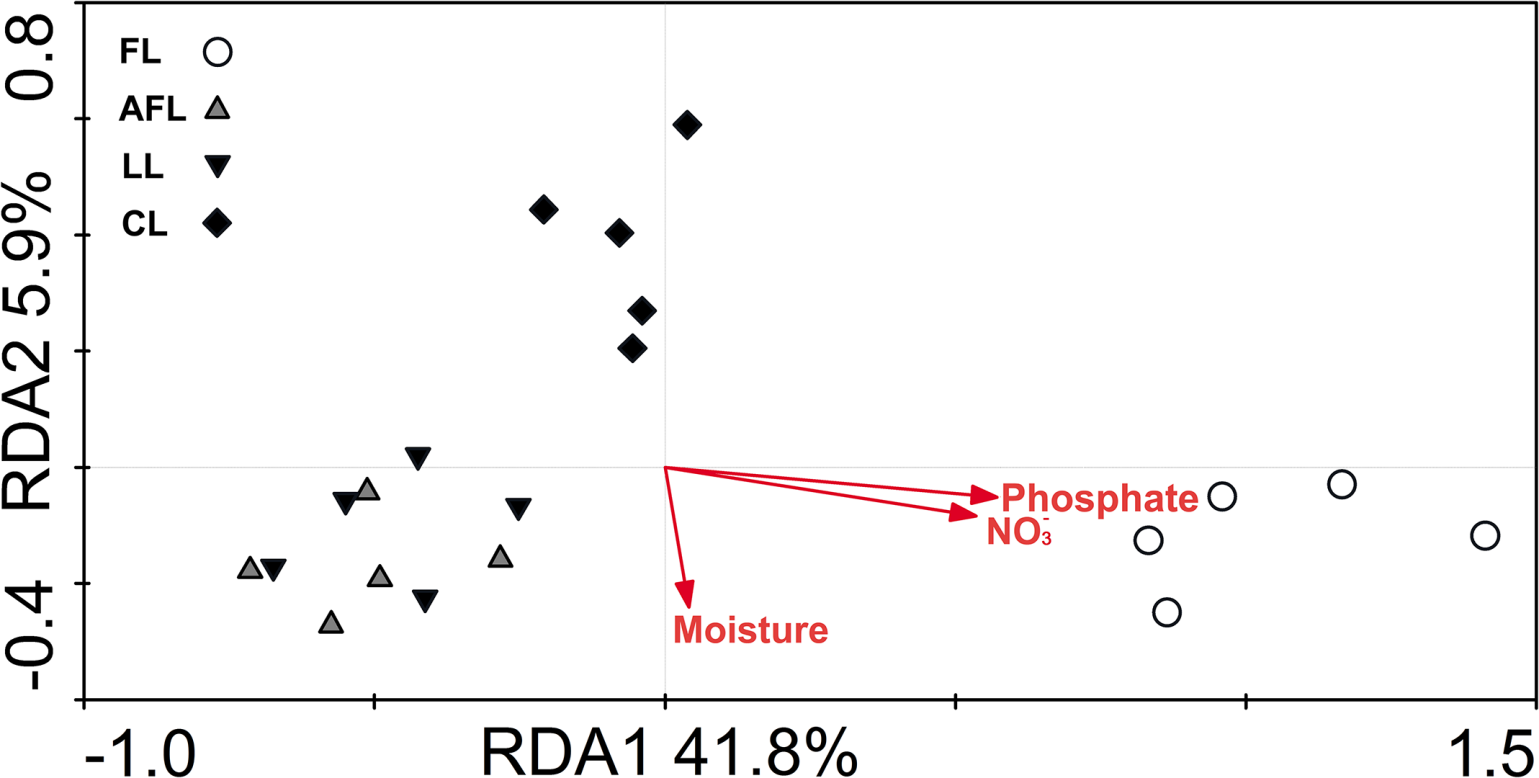
Redundancy analysis of the effect of soil parameters on ammonia-oxidizing bacterial communities using the terminal restriction fragment length polymorphism data. Only the soil parameters which significantly explained (*p* < 0.05 by 499 permutations) the ammonia-oxidizing bacterial community variation were shown. FL, farmland, AFL, abandoned farmland, LL, *Lolium perenne* L. land, CL, *Caragana korshinskii* Kom. land.

Totally, six terminal restriction fragments (tRFs) of AOB were detected from all the soil samples by the enzyme of *Msp* I (Fig 2). The relative abundance of different tRFs varied between restored and non-restored lands. Notably, the relative abundance of tRF 156 and 140 for the cropland was significantly lower than those for the restored land (Fig 2), while the relative abundance of tRF 256 for the cropland was significantly higher than those for the restored land (Fig 2). Both Cluster and PCA analyses revealed that the community composition of AOB was different between the restored and non-restored lands (S1 Fig). This was further confirmed by an ANOSIM of the restored and non-restored lands (Table 2). On average, higher values of ANOSIM's *R* were observed for restored and non-restored land comparisons (ANOSIM's *R* values 0.56–0.87, all *P* values < 0.05). Although less pronounced, there were also differences in community composition between restoration methods evidenced by the ANOSIM's *R* values for abandoned farmland and *Caragana korshinskii* Kom. land at 0.45 and *P* values < 0.05. Redundancy analysis was performed to identify the correlation between soil geochemical characteristics and variations in the community compositions of AOB. Nitrate and phosphate were the two main factors which influenced the community composition of AOB (Fig 3). In total, 42% of the variation of the AOB community could be significantly explained by nitrate and phosphate concentrations.

**Table 2:** ANOSIM R and P values for comparisons of ammonia-oxidizer communities using the terminal restriction fragment length polymorphism data.

	AOB		AOA	
	*R* value	*P* value	*R* value	*P* value
FL-AFL	0.87	0.01	0.12	0.08
FL-LL	0.82	0.01	-0.14	0.85
FL-CL	0.56	0.001	0.14	0.10
AFL-LL	0.04	0.30	0.27	0.04
AFL-CL	0.45	0.04	0.57	0.02
LL-CL	0.24	0.07	0.04	0.30
Total	0.47	0.001	0.15	0.03

FL, farmland, AFL, abandoned farmland, LL, *Lolium perenne* L. land, CL, *Caragana korshinskii* Kom. land.

For ammonia oxidizing archaea, eight T-RFs were detected using the enzymes of *Mbo* I. Only slight differences in the community compositions of AOA between abandoned farmland and *Lolium perenne* L. land (*R* = 0.27, *P* = 0.04) and abandoned farmland and *Caragana korshinskii* Kom. land (*R* = 0.57, *P* = 0.02) were observed in ANOSIM analyses (Table 2).

### Diversity of AOB and AOA in different soils as revealed by clone library analysis

Four clone libraries were constructed with the *amoA* amplifications for AOB and AOA respectively. Thirty clones per library were selected for sequencing. All acquired sequences were subjected to *in silico* digestion analysis.

**Fig 4:**
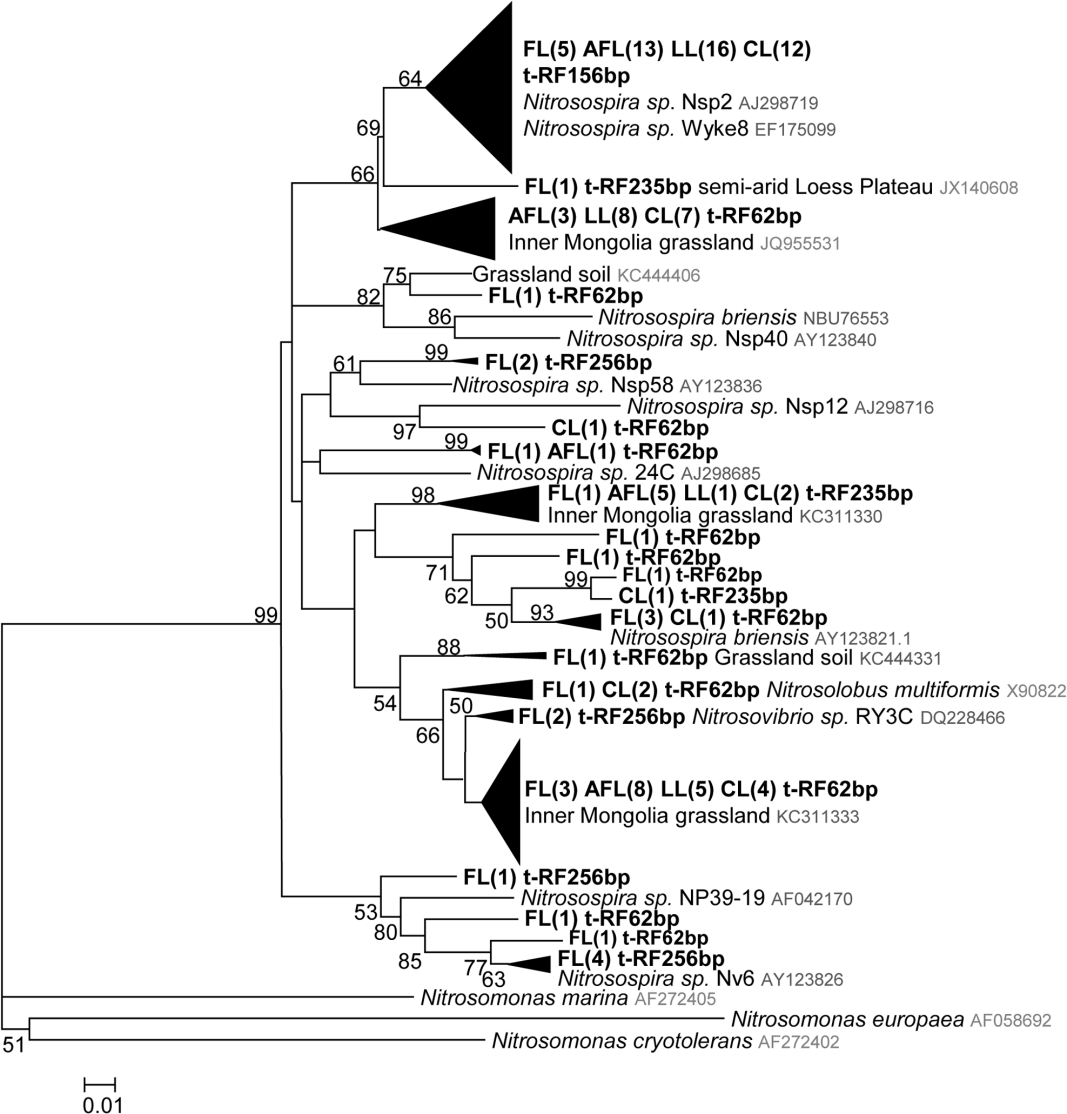
Neighbor-joining phylogenetic tree of the bacterial *amoA* sequences. The triangles represent compressed branches containing various sequences from different land cover as indicated; the vertical length of a triangle reflects the number of sequences, the horizontal length reflects the largest distance between sequences, and *in silico* tRF sizes are shown after the triangle or sequence number. Bootstrap values (> 50) are indicated at nodes. FL, farmland, AFL, abandoned farmland, LL, *Lolium perenne* L. land, CL, *Caragana korshinskii* Kom. land.

Four tRFs (tRF256, tRF235, tRF156 and tRF62) were detected from the clone library analysis for AOB (Fig 4). The relative abundance of dominant tRFs were comparable with their corresponding tRFs by tRFLP analysis (S1 Table). Interestingly, the sequences with tRF size of 256 bp were only detected for the cropland (Fig 4). Correspondingly, the highest relative abundance of tRF256 was found for cropland by tRFLP analysis (Fig 2). Among them, four sequences share high similarity (98% sequence identity) with the *amoA* gene sequence for *Nitrosospira* sp. Nv6 (Cluster 3b), and two sequences share 97% sequence identity with the *amoA* genes of *Nitrosospira* sp. Nsp58 (Cluster 2), and the other two sequences were similar to the *amoA* genes of *Nitrosovibrio* sp. RY3C (Cluster 3a.2 and Fig 4). More sequences with tRF size of 156 bp (13 for abandoned farmland, 16 for *Lolium perenne* L. land and 12 for *Caragana korshinskii* Kom. land) were detected for restored lands than the cropland (5 for farmland). All sequences with tRF size of 156 bp were closely (>95% sequence identity) related to the *amoA* genes of *Nitrosospira* sp. Nsp2 (Cluster 3a.1). Despite that the relative abundance of tRF62 was comparable between different soils, as suggested by both tRFLP and clone library analysis. Further sequence analysis revealed that 7 sequences (> 97% sequence identity) with tRF size 62 were only observed in the restored lands (S1 Table). Fewer OTUs were detected for restored soil with grass (8 for abandoned farmland and 7 for *Lolium perenne* L. land), while 20 OTUs were detected for the cropland (S1 Table).

For AOA, seven OTUs of eight tRFs were detected by the clone library analysis. The relative abundance of dominant OTUs was comparable between different soils (S4 Fig). However, OTU8 sharing 99% similarity with the *amoA* clone (HQ538571) from a nitrogen-rich wetland was only observed in *Lolium perenne* L. land and *Caragana korshinskii* Kom. land (S4 Fig and S2 Table). All the four dominant OTUs shared a relative low similarity with those *amoA* genes from cultivable strains, such as *Nitrososphaera viennensis* (S4 Fig and S2 Table).

## Discussion

Grain to Green Project has restored large areas of vegetation from cropland in semi-arid regions such as Inner Mongolia, resulting in several environmental benefits. In the present study, we explored the influence of three typical land restoration methods applied in semi-arid regions on the abundance and diversity of AOA and AOB in soils. Both the abundance and community composition of AOB were significantly different between the restored lands and the adjacent cropland, indicating that AOB, rather than AOA, was affected by land restoration in this type of ecosystem.

Soil pH is a prerequisite for determining the different responses of AOA and AOB to changes of land use [36]. The pH values in the present study ranged from 8.11 to 8.26, which are suitable for AOB. Laboratory cultures of AOB could only grow above neutral pH [20]. This finding is in agreement with results from previous researches [22, 24]. In the study of Nicol et al. [19], the bacterial transcript abundance of *amoA* increased with increasing pH and the highest pH recorded was only 7.5, which is lower than those in our study. Shen et al. [22] found that long-term fertilization significantly influenced the abundance and composition of AOB in a sandy loam soil with pH ranging from 8.3 to 8.7. In acidic soil conditions, however, Hu et al. [37] found that AOA was increasingly involved in ammonia oxidation after adding water to dryland soils. In a microcosm experiment with soil (pH 7.0), Denaturing Gradient Gel Electrophoresis analyses of both 16S *rRNA* genes and *amoA* genes suggested a dramatic response of AOA to different temperature in nitrifications [32]. The pH could possibly affect the bioavailability of ammonia, as NH_3_ would be ionized exponentially to NH_4_^+^ when pH decreased [19]. In the study by Khademi and Stroud [38], the ammonia transporter (*amtB*) on the membrane of *E*.*coli* could only conduct the transfer NH_3_ but not the protons. Recently, Offre et al. [39] discovered that the ammonia transporter for AOA was highly variable and clearly distinct from those for AOB. However, the specifics of how ammonia transporters of both ammonia oxidizers relate to pH remains to be explored.

Both the abundance and community composition of AOB significantly differed between the restored land and the cropland, for which the highest abundance of AOB and PNR activities were detected (Fig 1). This result suggested that bacterial ammonia oxidation was reduced after land restoration. In contrast to the restored land, urea and superphosphate were applied annually in the croplands. Mineral nitrogen fertilizations often lead to increased soil PNR and an abundance of AOB in alkaline soils [22, 24]. However, the effect of phosphate amendment on AOB has been rarely studied. Clone library analysis of *amoA* genes for AOB revealed that all sequences were *Nitrosospira-*like sequences (Fig 4). The dominance of *Nitrosospira-*like *amoA* sequences was also detected in several studies for terrestrial environments [22, 24, 40]. Seven of the AOB *amoA* gene sequences, detected exclusively in cropland soils, shared high similarity with the *amoA* genes of *Nitrosospira* sp. Nv6 (Fig 4). *Nitrosospira* sp. Nv6-like *amoA* genes were often detected in soils with elevated levels of mineral nitrogen fertilizer input [41, 42]. Compared with cropland, the proportion of *Nitrosospira* sp. Nsp2-like *amoA* gene sequences increased after restoration (Fig 4, S1 Table), suggesting that these populations possibly adapted to low level of ammonia in the restored soils. The soil water content was significantly lower in the scrubland soil than in other treatments (Table 1), possibly due to the high ratio of bare land in the *Caragana korshinskii* Kom. land, where the lowest PNR was detected. In a recent study by Hu et al. [37], water addition was found to enhance PNR and the abundance of AOA and AOB in dry ecosystems. However, the abundance of AOB in the *Caragana korshinskii* Kom land was significantly higher than those in the other two restored lands. The symbiotic of *Caragana korshinskii* Kom. with nitrogen-fixing microorganisms could increase the soil nitrogen source and possibly promote the growth of AOB [43]. Restoration associated changes with plant diversity and composition may have both directly and indirectly effects on AOB community. Plant could compete for ammonia with ammonia oxidizing microorganisms [6], while root exudates produced by plant also could boost the proliferation of ammonia oxidizing microorganisms [44]. However, the effect of legume plants on the abundance of AOB was not consistent [45]. Further studies on the effect of *Caragana korshinskii* Kom. on the AOB community are still needed.

In the present study, both the abundance and community composition of AOA were comparable between different soils, suggesting that land restoration might not affect the quality and quantities of microniches for AOA. However, real time qPCR results, which revealed that the abundance of AOA was much higher than that of AOB in all the soils studied (Fig 1), coincided with several studies [17, 22, 24]. These findings indicated that AOA was still predominant in these soils despite that theirs high pH values were thought to be unfavorable for AOA. Soil networks are highly heterogeneous and complex, possibly providing microniches suitable for the survival of AOA [46]. The oligotrophic life style, which only needs low levels of maintenance energy [47, 48], and the small cell size and small genome of most AOA [49] possibly contributed to its high abundance in soil as less energy is needed for its replication. The low water content in all the studied soils could reduce soil pore connectivity, which affects the diffusion of key factors. This might result in microniches with very limited ammonia. However, it is possibly that land restoration did not affect the quantities and qualities of microniches for AOA in this study.

Land restoration in Inner Mongolia, which was selected for its representation of semi-arid ecosystems, resulted in a significantly decreased abundance of AOB and ammonia oxidation activities, A significant difference in the community compositions of AOB was also observed between restored lands and the adjacent control, and AOB populations that possibly adapted to high concentration of inorganic N were negatively affected by the land restoration. Only a slight difference in AOB communities was observed between the restored land with the grass and with the shrub. These results indicate the potential role of GGP in reducing the emission of greenhouse gas N_2_O and in the leaching of nitrates into ground water.

## Supporting Information

S1 Dataset (XLS)

S1 Fig Effect of land restoration on ammonia-oxidizing bacterial community composition as assessed by Cluster (A) and PCA (B) scaling of terminal restriction fragment length polymorphism data. FL, farmland, AFL, abandoned farmland, LL, *Lolium perenne* L. land, CL, *Caragana korshinskii* Kom. land.
(TIF)

S2 Fig Terminal restriction fragment length polymorphism fingerprints of the archaea *amoA* gene fragments in different land cover. FL, farmland, AFL, abandoned farmland, LL, *Lolium perenne* L. land, CL, *Caragana korshinskii* Kom. land.
(TIFF)

S3 Fig Correlation of the PCA axes with NO_3_^-^ (a), phosphate (b), and moisture (c), respectively. (TIF)

S4 Fig Neighbor-joining phylogenetic tree of the archaea *amoA* sequences. The triangles represent compressed branches containing various sequences from different land cover as indicated, the vertical length of a triangle reflects the number of sequences, the horizontal length reflects the largest distance between sequences, and *in silico* tRF sizes are shown after the triangle or sequence number. Bootstrap values (> 50) are indicated at nodes. FL, farmland, AFL, abandoned farmland, LL, *Lolium perenne* L. land, CL, *Caragana korshinskii* Kom. land.
(TIF)

S1 Table OTU distribution of bacterial *amoA* gene sequences. (DOCX)

S2 Table OTU distribution of archaeal *amoA* gene sequences. (DOCX)
