# Supplementary figures and images for: Shifts in Abundance and Diversity of Soil Ammonia-Oxidizing Bacteria and Archaea Associated with Land Restoration in a Semi-Arid Ecosystem
Source: PLoS One. 2015 Jul 14;10(7):e0132879. doi: 10.1371/journal.pone.0132879 (PMC4501784; doi:10.1371/journal.pone.0132879)

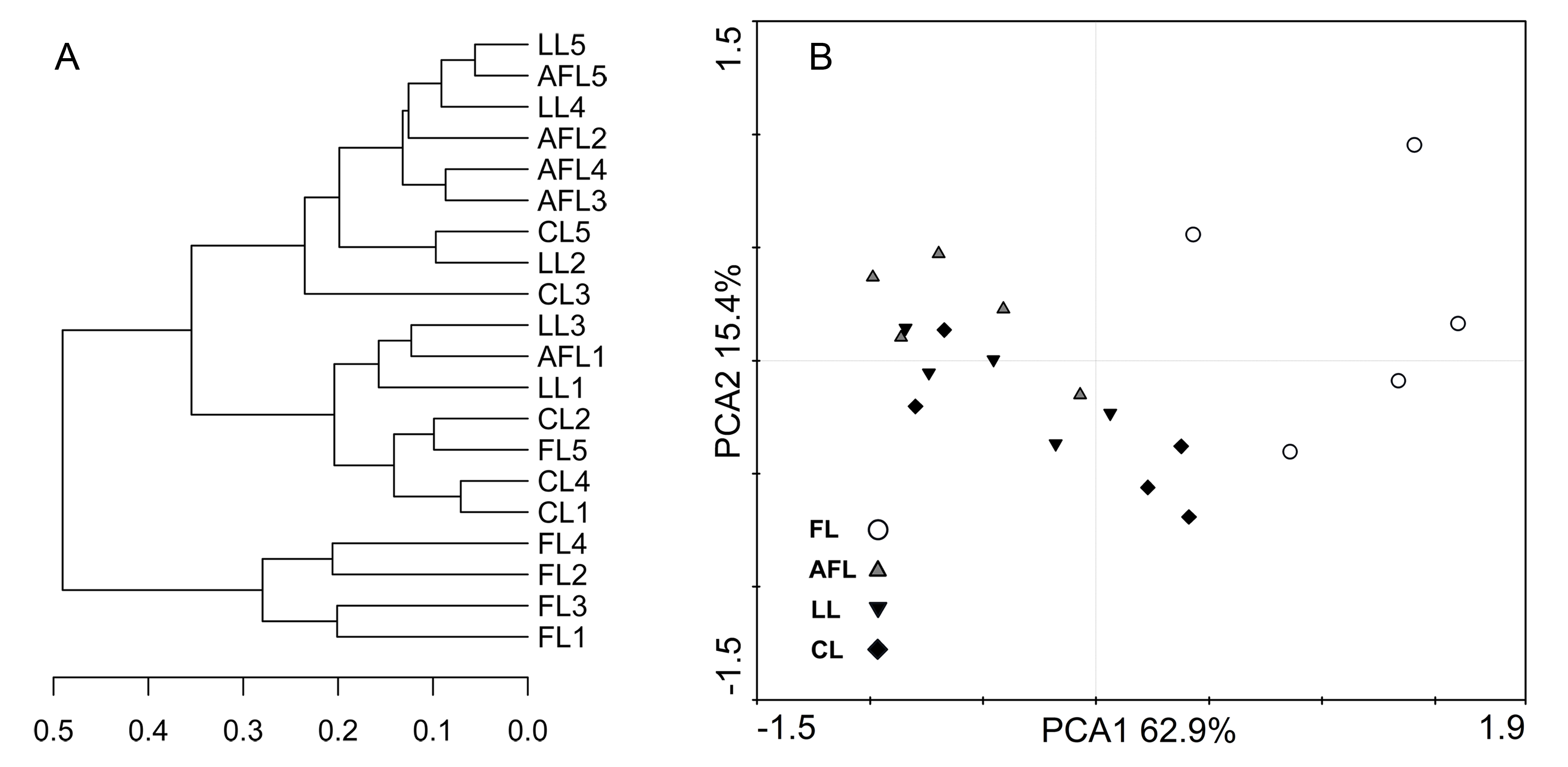

Supplement: S1 Fig — FL, farmland, AFL, abandoned farmland, LL, Lolium perenne L. land, CL, Caragana korshinskii Kom. land. (TIF) [file pone.0132879.s002.tif]

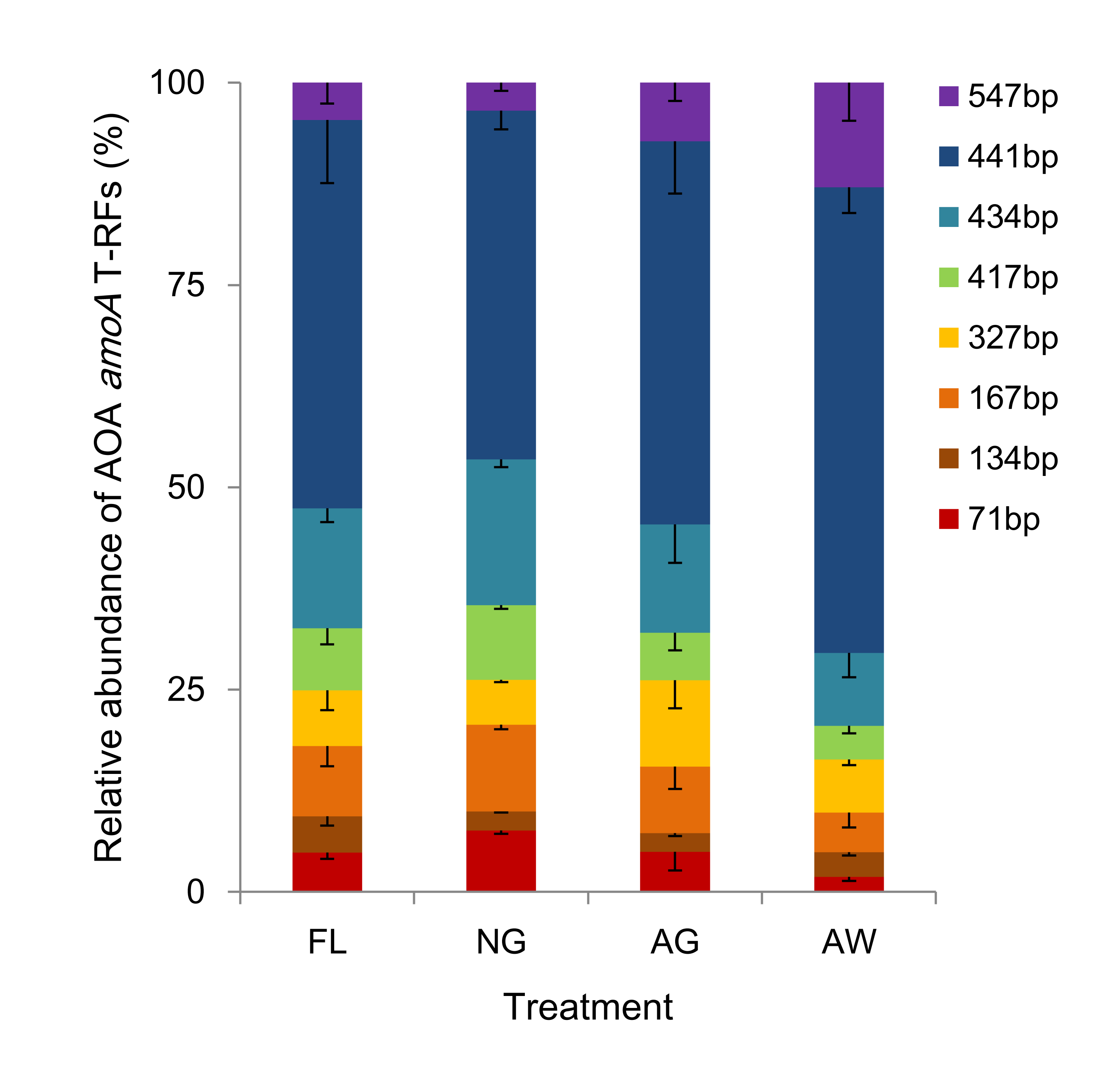

Supplement: S2 Fig — FL, farmland, AFL, abandoned farmland, LL, Lolium perenne L. land, CL, Caragana korshinskii Kom. land. (TIFF) [file pone.0132879.s003.TIFF]

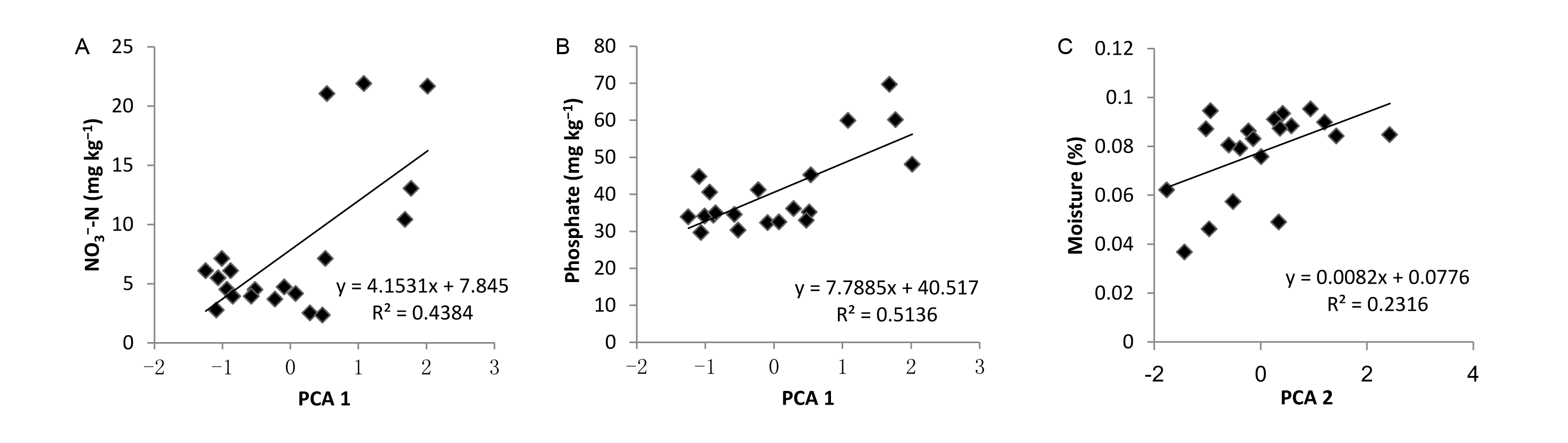

Supplement: S3 Fig — (TIF) [file pone.0132879.s004.tif]

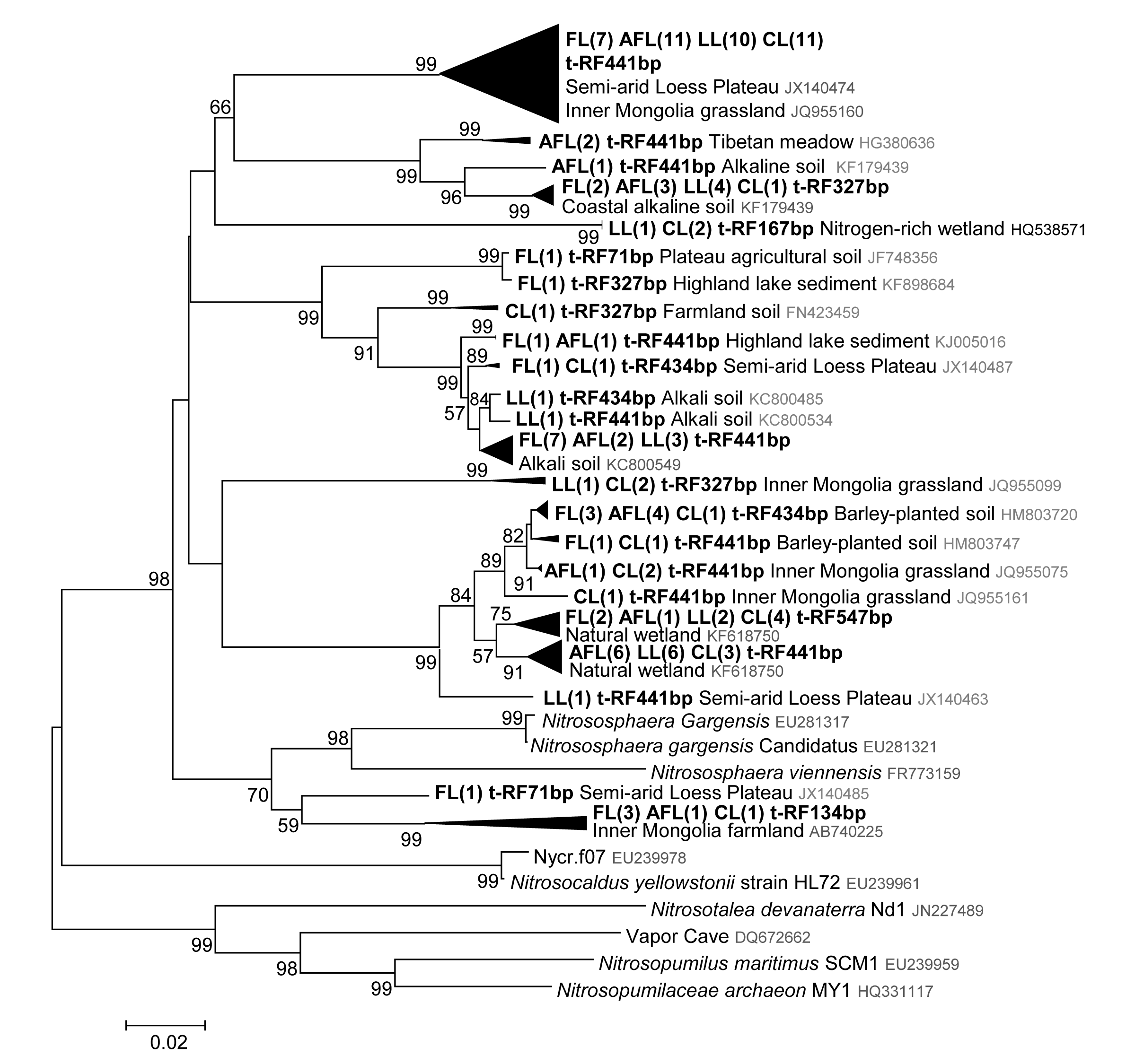

Supplement: S4 Fig — The triangles represent compressed branches containing various sequences from different land cover as indicated, the vertical length of a triangle reflects the number of sequences, the horizontal length reflects the largest distance between sequences, and in silico tRF sizes are shown after the triangle or sequence number. Bootstrap values (> 50) are indicated at nodes. FL, farmland, AFL, abandoned farmland, LL, Lolium perenne L. land, CL, Caragana korshinskii Kom. land. (TIF) [file pone.0132879.s005.tif]
