# Supplementary material for: Shifts in Abundance and Diversity of Soil Ammonia-Oxidizing Bacteria and Archaea Associated with Land Restoration in a Semi-Arid Ecosystem
Source: PLoS One. 2015 Jul 14;10(7):e0132879. doi: 10.1371/journal.pone.0132879 (PMC4501784; doi:10.1371/journal.pone.0132879)
Supplement: S1 Table — (DOCX) [file pone.0132879.s006.docx]

**S1 Table OTU distribution of bacteria *amoA* gene sequences.**

| **OTU** | **1** | **2** | **3** | **4** | **5** | **6** | **7** | **8** | **9** | **10** | | **11** | **12** | **13** | **14** | **15** | **16** |
| --- | --- | --- | --- | --- | --- | --- | --- | --- | --- | --- | --- | --- | --- | --- | --- | --- | --- |
| tRF | 156 | 62 | 62 | 235 | 62 | 256 | 62 | 62 | 62 | 62 | 235 | 235 | 256 | 62 | 62 | 62 | 62 |
| FL | 3 | 3 |  | 1 |  | 4 |  | 3 | 1 | 1 |  |  | 2 |  |  | 1 | 1 |
| AFL | 13 | 7 | 1 | 3 |  |  | 2 |  | 1 |  |  | 2 |  |  | 1 |  |  |
| LL | 14 | 5 | 4 | 1 | 4 |  |  |  |  |  |  |  |  |  |  |  |  |
| CL | 12 | 3 | 2 | 2 | 1 |  | 1 | 1 |  |  | 1 |  |  | 2 | 1 |  |  |
| **OTU** | **17** | **18** | **19** | **20** | **21** | **22** | **23** | **24** | **25** | **26** | **27** | **28** | **29** | **30** | **31** | **32** |  |
| tRF | 62 | 62 | 156 | 156 | 62 | 156 | 235 | 256 | 62 | 256 | 62 | 62 | 62 | 156 | 62 | 62 |  |
| FL | 1 | 1 |  |  |  | 1 | 1 | 1 | 1 | 1 |  |  | 1 | 1 | 1 |  |  |
| AFL |  |  |  |  |  |  |  |  |  |  |  |  |  |  |  |  |  |
| LL |  |  | 1 | 1 |  |  |  |  |  |  |  |  |  |  |  |  |  |
| CL |  |  |  |  | 1 |  |  |  |  |  | 1 | 1 |  |  |  | 1 |  |

FL farmland, AFL abandoned farmland, LL *Lolium perenne* L. land, CL *Caragana korshinskii* Kom. land.
