# Supplementary material for: Shifts in Abundance and Diversity of Soil Ammonia-Oxidizing Bacteria and Archaea Associated with Land Restoration in a Semi-Arid Ecosystem
Source: PLoS One. 2015 Jul 14;10(7):e0132879. doi: 10.1371/journal.pone.0132879 (PMC4501784; doi:10.1371/journal.pone.0132879)
Supplement: S2 Table — (DOCX) [file pone.0132879.s007.docx]

**S2 Table OTU distribution of archaeal amoA gene sequences.**

| **OTU** | **1** | **2** | **3** | | | **4** | **5** | **6** | **7** | **8** | **9** | | **10** | **11** | **12** | **13** |
| --- | --- | --- | --- | --- | --- | --- | --- | --- | --- | --- | --- | --- | --- | --- | --- | --- |
| tRF | 441 | 441 | 71 | 435 | 441 | 441 | 441 | 327 | 548 | 168 | 441 | 435 | 441 | 548 | 134 | 134 |
| FL | 2 |  | 1 | 2 | 1 | 7 | 3 | 2 | 2 |  | 1 |  |  |  | 2 |  |
| AFL | 4 | 6 |  | 4 | 1 | 2 | 4 | 2 | 1 |  | 1 |  |  |  |  | 1 |
| LL | 5 | 6 |  |  |  | 3 | 3 | 2 | 2 | 1 | 1 | 1 | 1 |  |  |  |
| CL | 9 | 3 |  | 1 | 3 |  |  | 1 | 2 | 2 |  |  | 2 | 2 |  | 1 |
| **OTU** | **14** | **15** | **16** | **17** | **18** | **19** | **20** | **21** | **22** | **23** | **24** | **25** | **26** | **27** | **28** |  |
| tRF | 441 | 435 | 441 | 327 | 441 | 327 | 441 | 441 | 441 | 441 | 441 | 441 | 441 | 327 | 327 |  |
| FL |  | 1 | 2 | 2 |  |  |  |  |  |  |  | 1 | 1 |  |  |  |
| AFL |  | 1 |  |  | 1 |  |  |  | 1 | 1 |  |  |  |  |  |  |
| LL | 2 |  |  |  |  | 1 | 1 |  |  |  | 1 |  |  |  |  |  |
| CL |  |  |  |  |  | 1 |  | 1 |  |  |  |  |  | 1 | 1 |  |

FL farmland, AFL abandoned farmland, LL *Lolium perenne* L. land, CL *Caragana korshinskii* Kom. land.
